# The Long-Term Changes in Dynamic Risk and Protective Factors Over Time in a Nationwide Sample of Dutch Forensic Psychiatric Patients

**DOI:** 10.3389/fpsyt.2021.737846

**Published:** 2021-09-16

**Authors:** Marija Janković, Geert van Boxtel, Erik Masthoff, Elien De Caluwé, Stefan Bogaerts

**Affiliations:** ^1^Department of Developmental Psychology, Tilburg University, Tilburg, Netherlands; ^2^Fivoor Science and Treatment Innovation (FARID), Rotterdam, Netherlands; ^3^Department of Cognitive Neuropsychology, Tilburg University, Tilburg, Netherlands

**Keywords:** forensic psychiatric patients, risk factors, protective factors, cluster B personality disorders, psychotic disorders, latent growth curve analysis, substance use disorder

## Abstract

The long-term changes of dynamic risk and protective factors have rarely been studied in forensic psychiatric patients. We utilized a latent growth curve analysis to investigate trajectories of risk and protective factors over time in all 722 male forensic psychiatric patients who were unconditionally released between 2004 and 2014 from any of 12 Dutch forensic psychiatric centers (FPCs). The study covered the period from juridical observation until unconditional release. Moreover, we investigated whether these trajectories differ between patients depending on their psychiatric diagnosis namely substance use disorders (SUD), psychotic disorders, and cluster B personality disorders (PDs). In addition, we also investigated whether SUD may influence changes in risk and protective factors in a group of psychotic and cluster B PDs patients, respectively. Overall, findings suggest that all changes in dynamic risk and protective factors could be depicted by two phases of patients' stay in the FPCs. Specifically, most changes on dynamic risk and protective factors occurred at the beginning of treatment, that is, from the time of juridical assessment up to the time of unguided leave. Moreover, the moment of unguided leave could be considered the ‘turning point’ in the treatment of offenders. We also found that SUD and psychotic patients changed the most in the first phase of their stay, while cluster B PDs patients changed the most in the second phase. However, SUD did not modify changes in risk and protective factors in psychotic and cluster B PDs patients. These findings may help improve offender treatment and crime prevention strategies.

## Introduction

In recent years, there has been a growing interest in longitudinal research on changes in dynamic risk and protective factors in forensic psychiatric patients. Dynamic risk and protective factors can be defined as potentially changeable characteristics of individuals and their environments that are expected to increase (risk factors) or decrease (protective factors) the likelihood of recidivism after discharge (1, 2). Previous research has shown that they are moderately to strongly associated with reoffending (3, 4). Dynamic risk factors and to a lesser extent protective factors are essential to forensic correctional practice; they can help set reasonable goals for interventions that reduce the likelihood of reoffending [e.g., *criminogenic needs*; (1), or *primary goods*; (5)], determine whether meaningful progress is being made toward treatment goals, and inform risk management strategies (6, 7). For these reasons, dynamic risk and protective factors are now routinely evaluated in structured risk assessment tools (7–9), such as the Historical, Clinical, and Future–Revised [HKT-R [*Historisch Klinisch Toekomst–Revised*]; (10)]. The HKT-R is comparable with the HCR-20-V^3^ (9), which is the most widely used risk assessment tool in the world for assessing violent risk. The HKT-R is developed in the Netherlands and should be mandatorily used for all admitted forensic patients and prisoners to investigate the future risk of recidivism and changes in recidivism risk.

It has been suggested that repeated measurements of dynamic risk and protective factors provide better and more valuable information for treatment progress in forensic psychiatric patients than dual time-points [e.g., pre-and post-treatment; (11, 12)]. Although useful, pre-and post-treatment measurements can only be indicative of whether a significant linear change in risk and protective factors has occurred, for instance, from admission to the forensic clinic until unconditional release (8). In contrast, multiple time-points allow for the measurement of different patterns and trajectories of change (11), which can offer a better understanding of treatment progress and an opportunity for forensic practitioners to adjust the treatment of offenders if needed.

Although the great importance of longitudinal research on the changeability of dynamic risk and protective factors has been recognized by many scholars, the trajectories of these factors during forensic treatment have so far rarely been investigated (7, 13). One reason for this is that the length of stay in forensic clinics is usually very long, thus collecting repeated measurements can be quite intensive and time-consuming. Especially for the forensic healthcare professionals who work with this challenging group of patients (14), collecting data at multiple time points can considerably increase their workload. Another reason has to do with obtaining sufficient statistical power due to the specificity of high-risk psychiatric patients staying in secure forensic facilities. For example, forensic psychiatric patients are very often unwilling to participate in a study or do not take the research seriously; there is also a high drop-out rate among patients, along with their limited understanding of the items, and a tendency for socially desirable answers (13).

Nevertheless, some studies have documented longitudinal trajectories of dynamic risk factors over time. In one such study, Douglas et al. (15) reported a linear decrease of dynamic risk factors over four time points, while a similar study detected no significant changes over time (12). However, both studies used very small samples of forensic patients and employed different risk assessment instruments. Recently, long-term trajectories related to the change of dynamic risk factors have been investigated in a relatively large sample of Dutch forensic psychiatric patients. The results showed a significant linear decrease in dynamic HKT-R risk factors, from judicial assessment until unconditional release [i.e., over five time points; (13)]. However, due to inconsistencies in findings in these few studies and scarcity of empirical evidence, further research is needed to better understand longitudinal changes in dynamic risk and protective factors in forensic psychiatric patients.

Moreover, all of these previous studies were based on aggregated data (i.e., a scale score) that included both risk and protective factors in the same measurement. Therefore, no conclusions can be drawn about changes in dynamic risk and protective factors separately. This emphasized the need for measuring more specific and detailed changes in dynamic risk and protective factors, for example, at a level with a small number of comparable factors. Therefore, the present study aimed to investigate changes in the HKT-R factors during treatment by examining trajectories of both the clinical scale (based on all the 14 HKT-R clinical factors) and the more fine-grained risk and protective subscales in a large nationwide sample of Dutch forensic psychiatric patients covering a period of ~9 years of institutional stay (i.e., five time points: from juridical observation until unconditional release). See Supplementary Table S1 in the Supplementary Materials for more details on the individual HKT-R factors. The clinical scale was expected to decrease significantly from juridical observation until unconditional release.

Furthermore, it is important to underline that the patients in high-security forensic psychiatric institutions differ in terms of dynamic risk and protective factors, which may be partly attributed to their diverse psychiatric diagnoses (16, 17). Substance use disorders (SUD) and psychotic disorders are particularly common clinical disorders in these patients, as are cluster B personality disorders [PDs; (18–20)]. These disorders have been shown to reinforce violent behavior and are important predictors of recidivism (6, 21, 22).

SUD is defined as a problematic pattern of using substances that leads to clinically significant impairment in daily life or distress (23). The odds of criminal behavior are three to four times higher in SUD patients compared to non-SUD patients (24). Kraanen et al. (25) found that 61.5% of violent offenders were diagnosed with SUD, while 29.9% were intoxicated during the offense. Substance use may lead to disinhibition making aggression more likely (6). In addition, patients with SUD are more likely to have difficulties in areas such as family relationships, employment, legal matters, housing, and health (20), which can also indirectly increase the risk of recurrence of criminal behavior. Furthermore, patients with SUD are considered difficult to treat because of their propensity for extreme emotional reactions and the difficulty of engaging them until abstinence is achieved. Research has shown that forensic patients who withdraw from treatment are more likely to use alcohol and/or drugs during treatment than patients who do not withdraw from treatment (26). Apart from that, there is also a high rate of comorbid psychiatric diagnoses in patients with SUD of which the most frequent are psychotic disorders (27), and cluster B PDs (28). Thus, this may further worsen the response and outcome of treatment (29).

The most common psychotic disorder is schizophrenia. Schizophrenia and other psychotic disorders are severe mental disorders characterized by the presence of delusions, hallucinations, paranoia, disorganized thinking (speech), grossly disorganized or abnormal motor behavior (including catatonia), and negative symptoms (23). People who experience these symptoms may appear to have lost contact with reality. Research has shown that patients with psychotic disorders are more likely to display dynamic risk factors, such as hostile behavior, poor impulse control, recent drug use, alcohol, and substance misuse, and non-compliance with medication and psychological therapies [for a review, see Ref. (30)]. In addition, untreated psychotic symptoms, often in combination with paranoia, are one of the main risk factors for violent behavior in psychotic patients (31). However, higher levels of psychopathy have been claimed to adversely influence treatment responsiveness (32) and 33% of forensic patients suffering from psychotic disorders are considered to be treatment resistant (33, 34). The presence of comorbid SUD in psychotic patients may even aggravate illness symptoms (35), leading to a poorer treatment prognosis. Psychotic patients with comorbid SUD are also more prone to medication non-compliance and generally have a higher risk of violent behavior than psychotic patients without comorbid SUD (36, 37).

Likewise, patients with cluster B PDs are more likely to reject treatment than seek it (38). Cluster B PDs include antisocial, borderline, histrionic, and narcissistic PDs. A defining characteristic of these disorders is a consistent pattern of disregard for and violation of the rights of others (23). People with cluster B PDs experience problems with emotion regulation, impulsivity and interpersonal conflicts (21, 38), and are characterized by a lack of empathy (39). The latter represents one of the main factors associated with serious and persistent criminal offending (40), while poor self-regulation and higher impulsivity are considered crucial in explaining criminal behavior according to the general theory of crime (41). In addition, many previous studies have often linked cluster B PDs to SUD, and antisocial behavior [e.g., (42–44)], i.e., factors that are also significant predictors of violent reoffending (1).

To summarize, patients with SUD, psychotic disorders or cluster B PDs are less likely to respond adequately to treatment and are more likely to recidivate after release from high-security forensic psychiatric institutions than patients without these disorders. It could be that these patients make less progress on dynamic risk and protective factors during forensic treatment, making them more likely to recidivate after release. Although a number of studies have contributed to a better understanding of specific risk and protective factors related to violence and recidivism in SUD, psychotic and cluster B psychiatric patients, to date, no studies have examined how risk and protective factors change during treatment in these patients. Therefore, in this study, we investigated whether changes in the clinical scale, and the risk and protective subscales over time are dependent on SUD, psychotic disorders, and cluster B PDs. It was expected that SUD, psychotic, and cluster B PDs patients would show less decrease on risk factors and less increase on protective factors over time than patients without these mental conditions. In addition, we also investigated whether SUD may influence changes in these factors in psychotic and cluster B PDs patients. It was hypothesized that psychotic and cluster B PDs patients with comorbid SUD would have a poorer treatment outcome than psychotic and cluster B PDs patients without comorbid SUD.

## Methods

### Participants

The original study sample consisted of all forensic psychiatric patients (*n* = 815) who were unconditionally released between 2004 and 2008 (*n* = 347, 8.6% female) and between 2009 and 2014 (*n* = 468, 13.5% female) from any of the 12 Dutch forensic psychiatric institutions. Female patients (*n* = 93, 11.4%) were excluded from this study because the sample size was too small for the intended statistical analysis. Therefore, the final sample comprised a total of 722 male patients. Of these 12 forensic institutions, there are six Dutch forensic psychiatric centers (FPCs), five forensic psychiatric clinics (FPKs) and one center for transcultural psychiatry (CTP)^1^. These institutions treat convicted offenders who have committed a serious crime caused by a severe mental illness or a personality disorder and are not held, or just partly, accountable for their offenses (45). Depending on the required treatment intensity and the estimated risk of recidivism (low, low to medium, medium, medium to high, high), these patients are placed by the judge in FPCs, which is a maximum secured institution, or FKPs or CTP, which are also secured institutions, but the security level is not as high as in the FPC.

### Procedure

The data were obtained from the electronic patient files with thorough descriptions of the background and criminal history of the patients, risk assessment scores, psychiatric reports and diagnoses, treatment plans, leave requests, and prolongation advice. Psychiatric diagnoses were based on the *Diagnostic and Statistical Manual of Mental Disorders* [4th ed., text rev. [DSM-IV-TR]; (46)] which was in use during the period for which the data were retrieved. Trained psychologists coded the HKT-R retrospectively for each patient based on available file information. The present study concerned the measurements of the HKT-R at five time points. The first time point (T1) refers to the scores obtained at the time of juridical assessment (performed by a psychiatrist and psychologist). The second time point (T2) refers to the scores after the first 12 months of the stay in the FPC. The third time point (T3) relates to the scores before the first unguided leave, which means that patients can leave the institution for a short period (e.g., half a day) without supervision. The fourth time point (T4) refers to the scores before conditional leave, which means that patients can live outside the institution but are still supervised by the correctional services. Finally, the fifth time point (T5) relates to the scores before unconditional release, which means that rules and agreements are no longer imposed and the patients are no longer under the supervision of correctional services. All data were anonymized and could not be traced back to individual patients. Information about violent recidivism rates has been obtained from the Dutch Ministry and Security of Justice. Forensic psychiatric patients who were released between 2004 and 2008 had been tracked from discharge until July 11, 2011, while patients released between 2009 and 2014 had been followed from discharge until June 20, 2018. The study has been approved by the Scientific Research Committee of the FPC Kijvelanden, the Dutch Ministry of Security and Justice, the 12 directors of the forensic institutions included in this study and the Ethical Review Board of Tilburg University.

### Measures

#### Risk and Protective Factors

Risk and protective factors were assessed using the risk assessment instrument HKT-R (10). The HKT-R is a structured professional risk assessment tool for assessing the risk of future violent and general recidivism in forensic psychiatric patients after release. The tool consists of three distinct domains comprising 12 historical factors, 14 clinical factors and seven future factors. Historical factors are static, irreversible and untreatable and refer to the offender's personal history up to the time of arrest for the current forensic psychiatric index offense (the offense that led to the conviction). Clinical and future factors are potentially changeable and therefore treatable. Clinical factors refer to the offender's behavior in the last 12 months, while future factors refer to the assessment of potential risks that may arise after release from the FPC (e.g., stressful circumstances, living arrangements and work situation). In this study, we used the 14 clinical indicators because the study covered the period of treatment. For more details on the individual HKT-R factors, see Supplementary Table S1 in the Supplementary Materials.

The clinical indicators were rated on a five-point Likert scale ranging from 0 = *no risk* to 4 = *high risk*. First, the *clinical scale* was created as an average score of the 14 clinical HKT-R indicators, where higher scores indicate a higher risk for recidivism. Moreover, to create the risk and protective subscales, we divided the clinical items into seven risk and seven protective factors as has been done in previous research (8). However, before creating the protective subscale, we reversely coded the protective factors such that 0 = *no protection* and 4 = *high protection*, while the coding of the risk factors remained unchanged (0 = *no risk* and 4 = *high risk*). In the next step, we applied exploratory factor analysis to validate the factor structure of these two subscales. In line with the study of Bogaerts et al. (8), factor analysis on the risk subscale revealed a one-factor solution (see Supplementary Table S2 in the Supplementary Materials). Hence, the *risk subscale* was created as an average score of the following seven risk items: psychotic symptoms, addiction, impulsivity, antisocial behavior, hostility, violation of terms and influence by risky network members. Factor analysis on the protective subscale, however, revealed a two-factor solution and we, therefore, split the protective subscale into a subscale referring to *protective awareness* and a subscale referring to *protective skills* (see Supplementary Table S3 in the Supplementary Materials). Average scores were also calculated for the *protective awareness subscale*, including problem insight, treatment compliance, and taking responsibility for the index offense, as well as for the *protective skills subscale*, including self-reliance, social skills, coping skills, and labor skills. In the current study, the internal consistency of the clinical scale was acceptable to good at all times of measurement, with Cronbach's alphas being α_T1_ = 0.85, α_T2_ = 0.82, α_T3_ = 0.76, α_T4_ = 0.74, and α_T5_ = 0.85.

#### SUD, Cluster B PDs and Psychotic Disorders

Diagnostic criteria for SUD, cluster B PDs and psychotic disorders were based on DSM-IV-TR (46). Diagnoses were determined by a psychiatrist in consultation with a clinical psychologist, taking into account all patients' information available at the time of admission to the FPC. SUD included excessive alcohol or drug use and was coded as 0 = *no diagnosis* and 1 = *diagnosis*. Cluster B PDs included antisocial personality disorder, borderline personality disorder, histrionic personality disorder and narcissistic personality disorder, and were coded as follows: 0 = *no diagnosis* and 1 = *diagnosis*. Finally, psychotic disorders included schizophrenia and related disorders and were coded such that 0 = *no diagnosis* and 1 = *diagnosis*.

### Statistical Analyses

All analyses were done using SPSS software version 25.0 (IBM Corp., Armonk, NY, USA) and the free software environment R (47). Prior to conducting the main analyses, the data were subjected to preliminary analyses regarding the assessment of missing data, identification of outliers, normality and multicollinearity. Data are considered to be not severely violated of normality if skewness is between −2 and +2, and kurtosis is between −7 and +7 (48, 49). Multicollinearity was measured by variance inflation factors (VIF) and tolerance; a VIF above 4.0 or tolerance below 0.2 signifies that multicollinearity might exist (49). Missing data were handled with full-information maximum likelihood (50). In addition, descriptive statistics of demographic and questionnaire data were computed. Furthermore, we utilized a latent growth curve analysis (LGCA) to investigate trajectories of the clinical scale as well as trajectories of the risk and two protective subscales over five time points. Additionally, it was investigated whether these trajectories are dependent on SUD, psychotic disorders and cluster B PDs. It was also investigated whether SUD may modify changes in risk and protective factors in patients with psychotic disorders and cluster B PDs, respectively. LGCA allows for investigating trajectories over time, characterized by the initial starting point (i.e., intercept) and change (i.e., slope). The LGCA was computed in R, using the lavaan package (51). Fit of the model was evaluated using the model chi-square statistic (*p* ≥ 0.05), comparative fit index (CFI; values ≥ 0.90), standardized root-mean-square residual (SRMR; <0.08), and root-mean-square error of approximation [RMSEA; <0.06; (52, 53)]. Finally, an analysis of variance (ANOVA) was performed to investigate the scores on the clinical scale as well as the risk and two protective subscales at five time points for SUD and non-SUD patients, psychotic and non-psychotic patients, and cluster B PDs and non-cluster B PDs patients.

## Results

### Sample Characteristics

Of the total sample of 722 male patients, 539 (74.6%) were born in the Netherlands and 183 (25.4%) were born elsewhere, such as in India and Suriname. The mean age at admission to the institution was 32.28 years (*SD* = 9.36, range = 17–79) with an average length of stay in the FPCs of 8.25 years (*SD* = 3.45, range = 1–26). The index offenses that led to admission included manslaughter (*n* = 244, 33.8%), moderate violence (*n* = 216, 29.1%), robbery (*n* = 170, 23.5%), severe violence (*n* = 113, 15.7%), murder (*n* = 111, 15.4%), sexual violence against adults (*n* = 100, 13.9%), arson (*n* = 88, 12.2%), and sexual violence against minors (*n* = 64, 8.9%). Patients could be convicted of multiple index offenses at the same time. At the beginning of treatment, the most frequent DSM-IV diagnoses were SUD (*n* = 310, 42.9%), PD not otherwise specified (*n* = 305, 42.2%), cluster B PDs (*n* = 199, 27.6%), and schizophrenia and other psychotic disorders (*n* = 178, 24.7%). These percentages do not count to exactly 100% as most patients had comorbid disorders. One hundred and four patients with cluster B PDs and 71 patients with psychotic disorders were also diagnosed with SUD. Within 2 years of release, 118 (16.6 %) patients reoffended violently. The means and standard deviations of clinical indicators of the HKT-R are displayed in Table 1.

**Table 1:** Means and standard deviations of clinical risk and protective factors.

	**T1**	**T2**	**T3**	**T4**	**T5**
**Variables**	***M (SD)***				
Clinical scale	1.79 (0.67)	1.42 (0.66)	0.96 (0.49)	0.82 (0.48)	0.68 (0.55)
Risk subscale	1.34 (0.75)	1.09 (0.75)	0.73 (0.54)	0.59 (0.49)	0.49 (0.55)
Protective awareness	1.28 (0.80)	1.93 (0.95)	3.19 (0.64)	2.80 (0.83)	3.22 (0.76)
Protective skills	2.33 (0.84)	2.47 (0.76)	2.87 (0.55)	3.04 (0.60)	3.03 (0.72)

*M, Mean; SD, Standard Deviation; T1, Judicial psychiatric assessment; T2, Admission to the clinic; T3, Unguided leave; T4, Conditional leave; T5, Unconditional leave*.

### Changes Over Time of the Clinical Risk and Protective Factors

**Figure 1:**
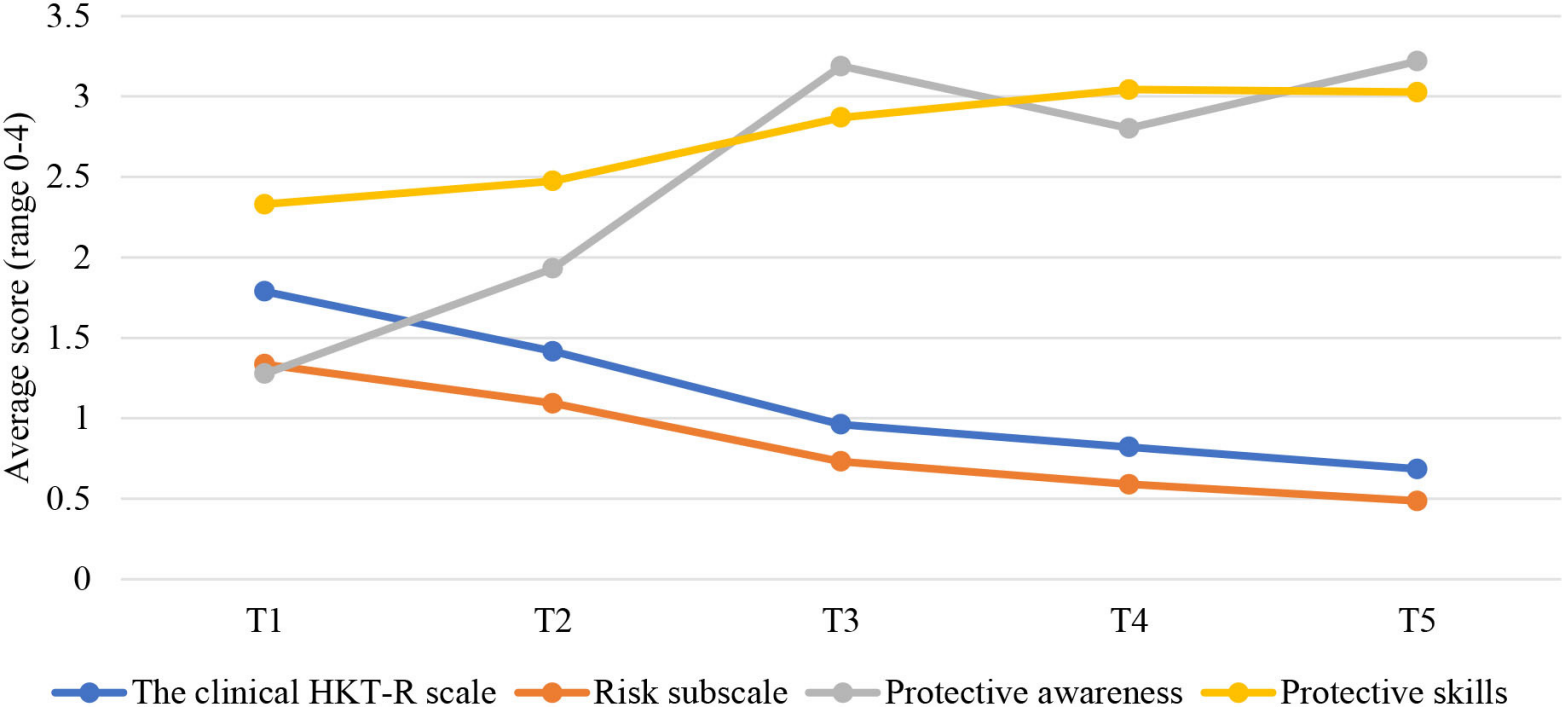
Changes over time in the clinical scale and the risk and two protective subscales. T1, Judicial psychiatric assessment; T2, Admission to the clinic; T3, Unguided leave; T4, Conditional leave; T5, Unconditional leave.

To investigate the trajectories of the clinical HKT-R scale, the risk subscale, and two protective subscales over five time points, LGCA was applied. These trajectories are shown in Figure 1. The assumptions of normality, no multicollinearity and no outliers were checked before conducting the LGCA. No violations of the assumptions were observed (for skewness and kurtosis, see Supplementary Table S4 in the Supplementary Materials).

#### Clinical Scale

First, we tested an unconditional model (i.e., without predictors) with a simple linear trajectory of the HKT-R clinical scale (consisting of 14 clinical indicators). This model fitted the data poorly, $\chi_{\left(10\right)}^{2}$ = 228.622, *p* < 0.001, CFI = 0.658, RMSEA = 0.174, and SRMR = 0.118. Consequently, an alternative so-called the linear piecewise model was tested. Linear piecewise models are used for modeling changes that deviate from a simple linear trajectory; when the rate of change during the specific time window differs from the rate of change during another time window (54). The simplest variant of the linear piecewise model is the two-phase model with two linear slopes and a single change point (55). In this model, the first linear slope represents the changes that occur during the first phase of the study, and the second linear slope describes the trajectories during the second phase. The change point represents the fixed time point where these two linear slopes are to be joined (54). Based on the plot (see Figure 1; clinical scale), the change point was assumed to be at T3, and therefore the unconditional two-phase linear piecewise model was tested. Compared to the single slope linear model, this model had a better fit, $\chi_{\left(6\right)}^{2}$ = 11.591, *p* = 0.07, CFI = 0.991, RMSEA = 0.036, and SRMR = 0.028. The mean of the intercept factor was 1.790, which closely corresponds with the observed mean of 1.789 at T1. The mean of the first slope factor was −0.406, *p* < 0.001, indicating that there was a significant decrease of ~0.406 on the clinical scale at each time point during the first phase of the study (T1–T3). The mean of the second slope factor was −0.145, *p* < 0.001, indicating that a level of the clinical scale continued to decline, but at a slower rate, of ~0.145 at each time point during the second phase of the study (T3–T5). The difference in slopes suggests that the rate of decline on the clinical scale was not constant throughout the entire stay in the FPCs. The rate of change was greater in the first phase than in the second phase. This was tested by constraining the two slope means to be equal, with the results showing that the slopes were significantly different, Δ$\chi_{\left(1\right)}^{2}$ = 155.6, *p* < 0.001. Finally, the variance of the intercept (0.258), and both slopes (0.045, 0.062) were significant, *p* < 0.001, showing the significant between-person variance of the initial score on the clinical scale and the slopes. The latter result indicates that the risk level of some patients decreased to a greater or lesser extent over time.

#### Risk Subscale

To gain more insight into detail-level changes, the clinical HKT-R indicators were split into a *risk* subscale and two protective subscales, one related to *protective skills* and the other to *protective awareness*. Examination of the single linear slope of the risk subscale resulted in a model that did not fit the data well, $\chi_{\left(10\right)}^{2}$ = 88.044, *p* < 0.001, CFI = 0.826, RMSEA = 0.104, and SRMR = 0.075. Hence, an alternative model was tested. Based on the plot (see Figure 1; risk subscale), we assumed that two linear segments joined at T3 would comprise the overall change process. Examining the two-phase unconditional linear piecewise model resulted in an excellent fit to the data, $\chi_{\left(6\right)}^{2}$ = 13.263, *p* = 0.04, CFI = 0.984, RMSEA = 0.041, and SRMR = 0.030. The mean of the intercept factor was 1.342, which closely corresponds with the observed mean of 1.335 at T1. The mean of the first slope factor was −0.299, *p* < 0.001, indicating that there was a significant decrease of ~0.299 on the risk subscale at each time point in the first phase of the stay in the FPCs (T1–T3). The mean of the second slope factor was −0.128, *p* < 0.001, indicating that a level of the risk scale continued to decline, but at a slower rate, of ~0.128 at each time point in the second phase of the stay (T3–T5). The rate decline on the risk subscale was not constant as evidenced by the difference in slopes; the rate of change was greater in the first phase than in the second phase. This hypothesis was tested by constraining the two slope means to be equal, with the results showing that the slopes were significantly different, Δ$\chi_{\left(1\right)}^{2}$ = 57.236, *p* < 0.001. Finally, the variance of the intercept (0.278), and both slopes (0.037, 0.048) were significant, *p* < 0.001, showing the significant between-person variance of the initial level on the risk subscale and the slopes.

#### Protective Skills

In addition, we also tested an unconditional model with a simple linear trajectory of the subscale protective skills. This model did not fit the data well, $\chi_{\left(10\right)}^{2}$ = 138.881, *p* < 0.001, CFI = 0.711, RMSEA = 0.134 and SRMR = 0.108. An alternative two-phase linear piecewise model was then tested. Based on the plot (see Figure 1; protective skills), we assumed that the rate of change on protective skills would be greater from T1 till T3, than from T3 till T5. Compared to the single linear trajectory model, this model had an acceptable fit, $\chi_{\left(6\right)}^{2}$ = 38.713, *p* < 0.001, CFI = 0.927, RMSEA = 0.087 and SRMR = 0.056. The mean of the intercept factor was 2.301, which corresponds to the observed mean of 2.330 at T1. The mean of the first slope factor was 0.280, *p* < 0.001, indicating that there was a significant increase of ~0.280 on the protective skills at each time point in the first phase of stay in the FPCs (T1–T3). The mean of the second slope factor was 0.092, *p* < 0.001, indicating that a level of the protective skills continued to increase, but at a substantially slower rate, of ~0.092 at each time point in the second phase of the stay (T3–T5). To test whether the rate of change was significantly greater in the first phase than in the second phase of stay, we constrained the two slope means to be equal. The results showed that the slopes were significantly different, Δ$\chi_{\left(1\right)}^{2}$ = 48.13, *p* < 0.001. Finally, the variance of the intercept (0.382), and both slopes (0.081, 0.092) were significant, *p* < 0.001, showing the significant between-person variance of the initial level on the protective skills and the slopes.

#### Protective Awareness

Finally, examining the single linear slope of the protective awareness subscale resulted in a model that did not fit the data well, $\chi_{\left(10\right)}^{2}$ = 903.931, *p* < 0.001, CFI = 0.001, RMSEA = 0.352 and SRMR = 0.488. Hence, an alternative piecewise model with a change point at T3 was tested. However, this alternative model poorly fitted the data, $\chi_{\left(6\right)}^{2}$ = 197.408, *p* < 0.001, CFI = 0.641, RMSEA = 0.210 and SRMR = 0.136 and therefore, the results cannot be interpreted. It is plausible that a piecewise model with three rather than two linear slopes would sufficiently capture the non-linear change of protective awareness. However, this more complex three-slope model could not be tested as we only had five time points and this analysis requires at least seven time points for identification (56).

### Patients With SUD vs. Patients Without SUD

The results showed that SUD significantly predicted trajectories over time, but only for the protective skills subscale. SUD was not significantly associated with the trajectories of the clinical scale and the risk subscale.

**Figure 2:**
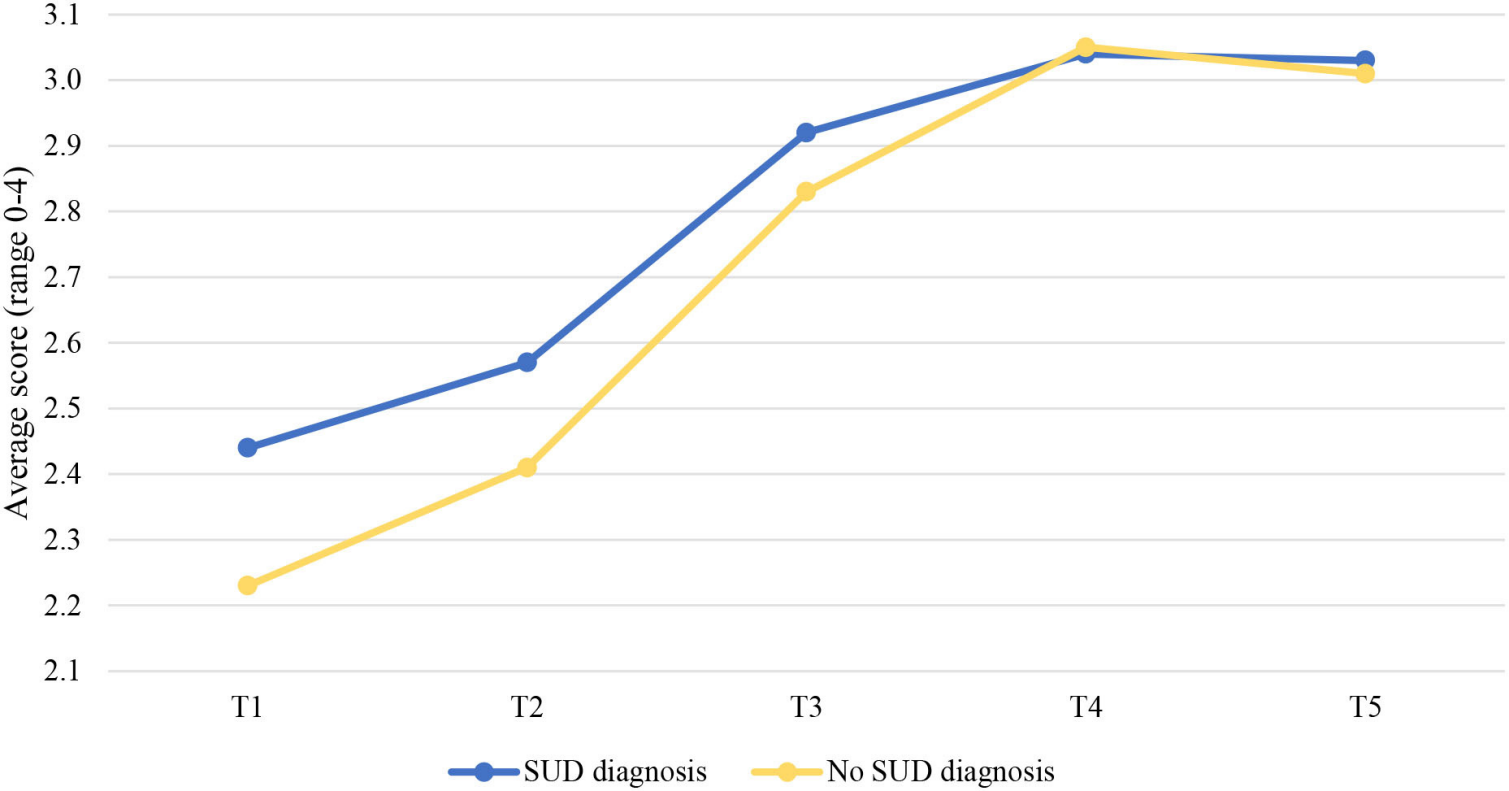
Changes over time in the protective skills subscale for patients with and without SUD. SUD, Substance use disorders; T1, Judicial psychiatric assessment; T2, Admission to the clinic; T3, Unguided leave; T4, Conditional leave; T5, Unconditional leave.

Examining the two-phase linear piecewise model of the protective skills subscale for patients with and without SUD resulted in an acceptable model fit, χ2_(8)_ = 38.849, *p* < 0.001, CFI = 0.933, RMSEA = 0.007 and SRMR = 0.048. However, SUD was only significantly associated with trajectories of the protective skills subscale in the first phase of residence in the FPCs (T1–T3), *b* = −0.066, *p* = 0.044. The level of the protective skills increased faster from T1 to T3 for SUD patients (*b* = 0.308 *p* < 0.001) compared to non-SUD patients (*b* = 0.242 *p* < 0.001; Figure 2). This hypothesis was tested by comparing a model with varying slopes for the two groups with a model with the same slopes. The results showed that the slopes were significantly different, Δχ2_(1)_ = 33.84, *p* < 0.001. We did not test differences in changes in the protective awareness subscale over time between these two groups of patients, as this model did not fit well in the present study and the results cannot be interpreted. Finally, differences in risk and protective factors between SUD and non-SUD patients, considering each time point, are displayed in Table 2.

**Table 2:** Differences in the means of clinical risk and protective factors of different subgroups of patients.

	**Psychotic disorders**		***F* test**	**Cluster B PDs**		***F* test**	**SUD**		***F* test**
**Variables**	**Diagnosis**	**No diagnosis**		**Diagnosis**	**No diagnosis**		**Diagnosis**	**No diagnosis**	
	***M (SD)***			***M (SD)***			***M (SD)***		
**Clinical scale**									
T1	2.21 (0.69)	1.66 (0.61)	*F*_(1,719)_ = 97.414^***^	1.88 (0.61)	1.75 (0.68)	*F*_(1,719)_ = 5.056^*^	1.75 (0.67)	1.82 (0.66)	*F*_(1,719)_ = 1.735
T2	1.60 (0.71)	1.36 (0.64)	*F*_(1,535)_ = 13.651^***^	1.49 (0.61)	1.38 (0.68)	*F*_(1,535)_ = 3.039	1.37 (0.65)	1.44 (0.67)	*F*_(1,535)_ = 1.450
T3	1.01 (0.54)	0.95 (0.48)	*F*_(1,639)_ = 1.444	1.04 (0.49)	0.93 (0.49)	*F*_(1,639)_ = 6.529^*^	0.92 (0.46)	0.99 (0.51)	*F*_(1,639)_ = 2.821
T4	0.91 (0.51)	0.78 (0.46)	*F*_(1,353)_ = 5.633^*^	0.88 (0.47)	0.80 (0.48)	*F*_(1,353)_ = 2.137	0.85 (0.48)	0.80 (0.48)	*F*_(1,353)_ = 0.675
T5	0.72 (0.52)	0.67 (0.57)	*F*_(1,716)_ = 0.854	0.71 (0.51)	0.68 (0.57)	*F*_(1,716)_ = 0.434	0.67 (0.53)	0.70 (0.57)	*F*_(1,716)_ = 0.617
**Risk subscale**									
T1	1.72 (0.82)	1.22 (0.69)	*F*_(1,716)_ = 59.624^***^	1.48 (0.71)	1.28 (0.76)	*F*_(1,716)_ = 9.839^*^	1.35 (0.74)	1.33 (0.76)	*F*_(1,716)_ = 0.160
T2	1.23 (0.80)	1.05 (0.72)	*F*_(1,531)_ = 6.132^*^	1.21 (0.70)	1.05 (0.76)	*F*_(1,531)_ = 5.085^*^	1.06 (0.72)	1.11 (0.77)	*F*_(1,531)_ = 0.625
T3	0.73 (0.57)	0.73 (0.53)	*F*_(1,638)_ = 0.013	0.85 (0.54)	0.68 (0.53)	*F*_(1,638)_ = 12.976^***^	0.74 (0.51)	0.73 (0.57)	*F*_(1,638)_ = 0.54
T4	0.63 (0.52)	0.57 (0.47)	*F*_(1,353)_ = 0.859	0.71 (0.48)	0.55 (0.48)	*F*_(1,353)_ = 7.330^*^	0.63 (0.49)	0.56 (0.49)	*F*_(1,353)_ = 1.582
T5	0.49 (0.54)	0.48 (0.55)	*F*_(1,716)_ = 0.023	0.52 (0.49)	0.47 (0.57)	*F*_(1,716)_ = 0.908	0.47 (0.53)	0.50 (0.56)	*F*_(1,716)_ = 0.340
**Protective awareness**									
T1	0.86 (0.65)	1.40 (0.80)	*F*_(1,705)_ = 60.062^***^	1.20 (0.68)	1.31 (0.84)	*F*_(1,705)_ = 2.462	1.26 (0.78)	1.29 (0.81)	*F*_(1,705)_ = 0.155
T2	1.67 (0.96)	2.02 (0.93)	*F*_(1,510)_ = 13.012^***^	1.85 (0.90)	1.97 (0.97)	*F*_(1,510)_ = 1.698	1.95 (0.99)	1.91 (0.92)	*F*_(1,510)_ = 0.157
T3	2.94 (0.67)	3.25 (0.62)	*F*_(1,633)_ = 25.045^***^	3.11 (0.68)	3.22 (0.63)	*F*(1,633)=3.267	3.22 (0.63)	3.16 (0.66)	*F*(1,633)=1.692
T4	2.61 (0.92)	2.88 (0.78)	*F*_(1,341)_ = 7.568^*^	2.77 (0.82)	2.81 (0.84)	*F*_(1,341)_ = 0.125	2.76 (0.80)	2.82 (0.85)	*F*_(1,341)_ = 0.217
T5	3.18 (0.73)	3.23 (0.76)	*F*_(1,704)_ = 0.660	3.18 (0.73)	3.24 (0.77)	*F*_(1,704)_ = 0.857	3.24 (0.71)	3.21 (0.79)	*F*_(1,704)_ = 0.342
**Protective skills**									
T1	1.92 (0.90)	2.45 (0.78)	*F*_(1,717)_ = 55.368^***^	2.34 (0.81)	2.33 (0.85)	*F*_(1,717)_ = 0.019	2.46 (0.83)	2.23 (0.83)	*F*_(1,717)_ = 13.544^***^
T2	2.29 (0.80)	2.53 (0.74)	*F*_(1,524)_ = 9.823^*^	2.46 (0.74)	2.48 (0.77)	*F*_(1,524)_ = 0.062	2.57 (0.78)	2.41 (0.75)	*F*_(1,524)_ = 0.5.917^*^
T3	2.79 (0.62)	2.89 (0.53)	*F*_(1,633)_ = 3.313	2.87 (0.54)	2.87 (0.55)	*F*(1,633)=0.002	2.92 (0.52)	2.83 (0.57)	*F*(1,633)=3.740
T4	2.95 (0.65)	3.08 (0.58)	*F*_(1,350)_ = 3.632	3.04 (0.58)	3.04 (0.61)	*F*(1,350)=0.003	3.04 (0.60)	3.05 (0.60)	*F*(1,350)=0.032
T5	2.97 (0.67)	3.05 (0.74)	*F*_(1,706)_ = 1.535	3.02 (0.72)	3.03 (0.72)	*F*_(1,706)_ = 0.008	3.03 (0.72)	3.01 (0.75)	*F*_(1,706)_ = 0.839

*M, Mean; SD, Standard Deviation; PD, Personality disorder; SUD, Substance use disorders; T1, Judicial psychiatric assessment; T2, Admission to the clinic; T3, Unguided leave; T4, Conditional leave; T5, Unconditional leave;*

* *p < 0.05,*

*** *p < 0.001*.

### Patients With Psychotic Disorders vs. Patients Without Psychotic Disorders

**Figure 3:**
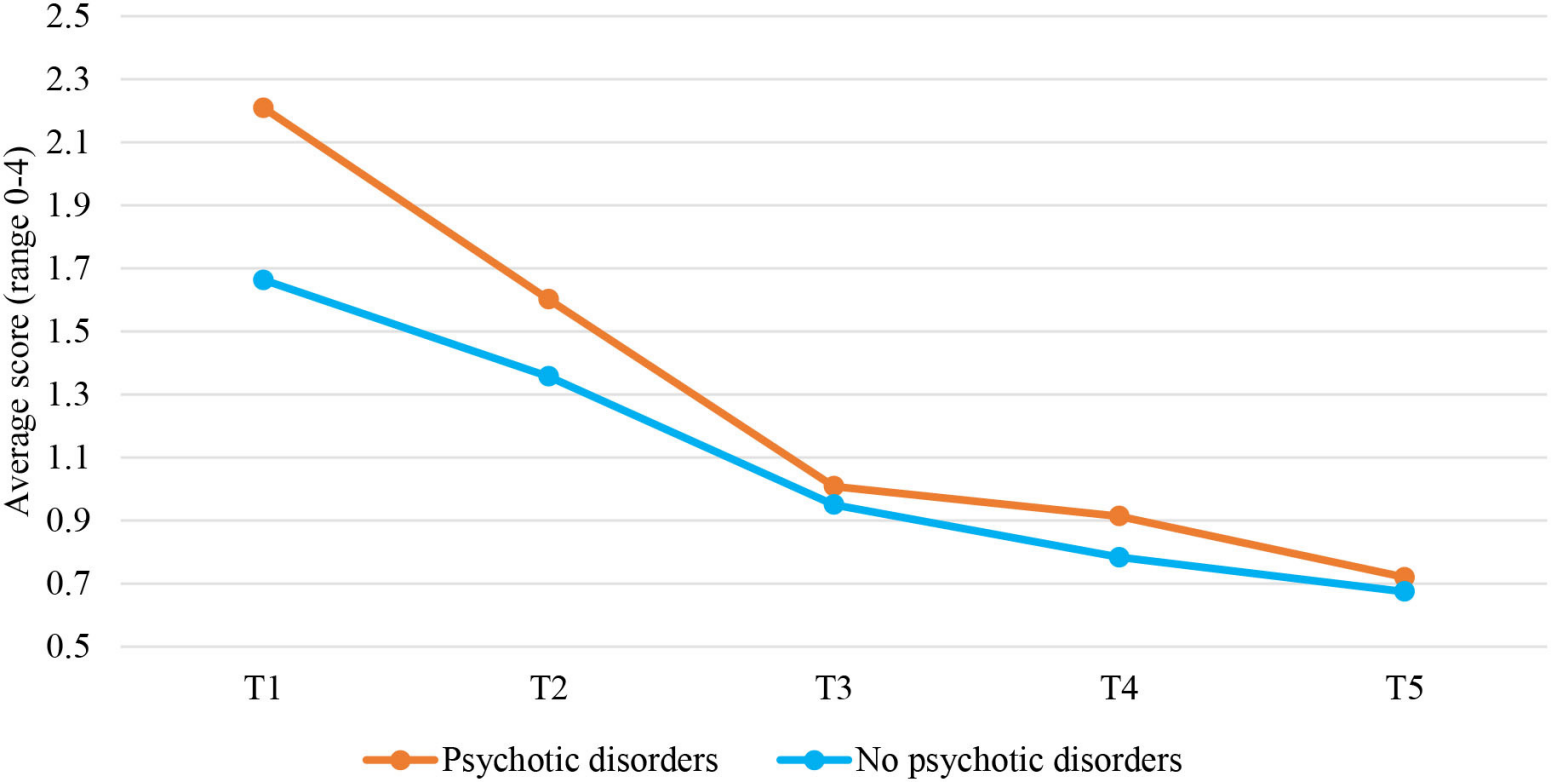
Changes over time in the clinical scale for patients with and without psychotic disorders. T1, Judicial psychiatric assessment; T2, Admission to the clinic; T3, Unguided leave; T4, Conditional leave; T5, Unconditional leave.

Investigating the two-phase linear piecewise model of the clinical scale for patients with psychotic disorders and those without resulted in a model that still fitted the data well, $\chi_{\left(8\right)}^{2}$ = 16.266, *p* = 0.039, CFI = 0.989, RMSEA = 0.038, and SRMR = 0.026. Psychotic disorders significantly predicted trajectories of the clinical scale, but only in the first phase of stay in the FPCs (T1–T3), *b* = 0.219, *p* < 0.001. The level of the clinical scale decreased at a greater rate from T1 to T3 for patients with psychotic disorders (*b* = −0.575, *p* < 0.001) compared to patients without these disorders (*b* = −0.356 *p* < 0.001; Figure 3). This hypothesis was tested by comparing a model with varying slopes for the two groups to a model with the same slopes. The results showed the slopes were significantly different, Δ$\chi_{\left(1\right)}^{2}$ = 89.967, *p* < 0.001.

**Figure 4:**
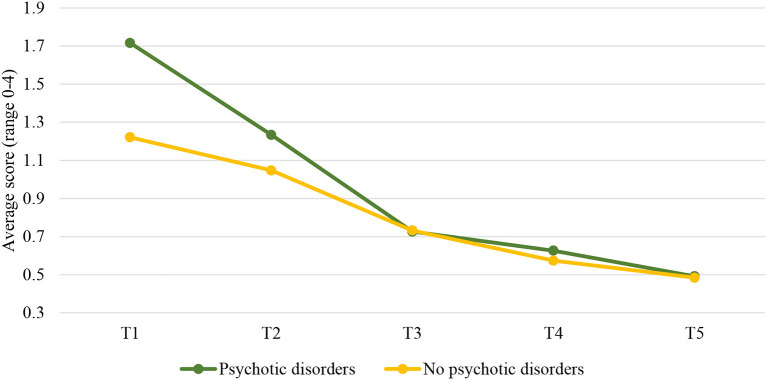
Changes over time in the risk subscale for patients with and without psychotic disorders. T1, Judicial psychiatric assessment; T2, Admission to the clinic; T3, Unguided leave; T4, Conditional leave; T5, Unconditional leave.

Likewise, investigating the two-phase linear piecewise model of the risk subscale for patients with and without psychotic disorders resulted in a well-fitting model, $\chi_{\left(8\right)}^{2}$ = 16.076, *p* = 0.041, CFI = 0.984, RMSEA = 0.037, and SRMR = 0.027. Psychotic disorders significantly predicted trajectories of the risk subscale, but only in the first phase of the patients' stay (T1–T3), *b* = 0.228, *p* < 0.001. The level of the risk scale decreased at a greater rate from T1 to T3 for patients with psychotic disorders (*b* = −0.475, *p* < 0.001) compared to those without these disorders (*b* = −0.247 *p* < 0.001; Figure 4). This hypothesis was tested by comparing a model with varying slopes for the two groups to a model with the same slopes. The results showed the slopes differed significantly, Δ$\chi_{\left(1\right)}^{2}$ = 52.034, *p* < 0.001.

**Figure 5:**
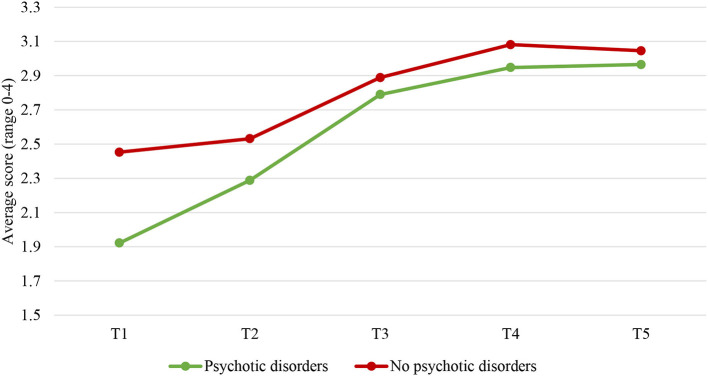
Changes over time in the protective skills subscale for patients with and without psychotic disorders. T1, Judicial psychiatric assessment; T2, Admission to the clinic; T3, Unguided leave; T4, Conditional leave; T5, Unconditional leave.

The two-phase linear piecewise model of the protective skills for patients with psychotic disorders and those without was also tested. The model had a good fit to the data, $\chi_{\left(8\right)}^{2}$ = 40.913, *p* < 0.001, CFI = 0.934, RMSEA = 0.075, and SRMR = 0.049. Psychotic disorders significantly predicted trajectories of the protective skills, but only in the first phase of the patients' stay in the FPCs (T1–T3), *b* = −0.206, *p* < 0.001. The level of protective skills increased at a greater rate for patients with psychotic disorders (*b* = 0.438, *p* < 0.001) compared to patients without these disorders (*b* = 0.232 *p* < 0.001; Figure 5). This hypothesis was tested by comparing a model with varying slopes for the two groups to a model with the same slopes. The results showed that the slopes were significantly different, Δ$\chi_{\left(1\right)}^{2}$ = 22.201, *p* < 0.001. Of note, we did not test whether psychotic and non-psychotic patients differ in scores on the protective awareness subscale over time because this model had a poor fit to the data in our study and results should not be interpreted. Between-group differences in the risk and protective factors at both scale and subscale levels for each time point are displayed in Table 2.

Finally, it was also tested whether SUD may modify the changes in the clinical scale and the risk and protective skills subscale over time in a group of psychotic patients. However, the results showed no significant differences in these changes between psychotic patients with and without SUD.

### Patients With Cluster B PDs vs. Patients Without Cluster B PDs

The results showed that cluster B PDs significantly predicted trajectories over time, but only for the risk subscale. Cluster B PDs were not significantly associated with the trajectories of the clinical scale and the protective skills subscale.

**Figure 6:**
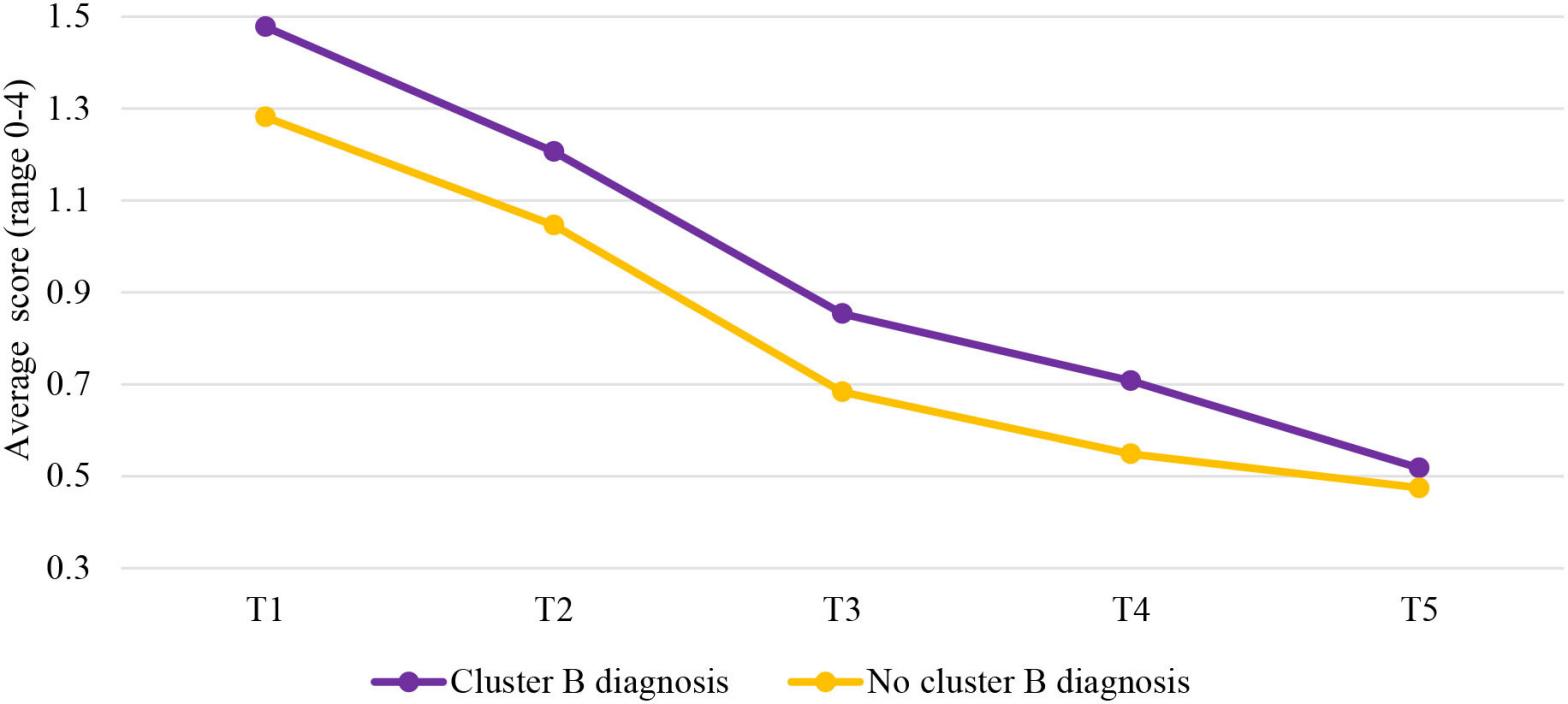
Changes over time in the risk subscale for patients with and without cluster B diagnosis. T1, Judicial psychiatric assessment; T2, Admission to the clinic; T3, Unguided leave; T4, Conditional leave; T5, Unconditional leave.

Examining the two-phase linear piecewise model of the risk subscale for patients with cluster B diagnosis and those without resulted in the model that fitted the data well, $\chi_{\left(8\right)}^{2}$ = 14.002, *p* = 0.082, CFI = 0.987, RMSEA = 0.032, and SRMR = 0.027. Cluster B PDs were only significantly associated with trajectories of the risk subscale in the second phase of the stay in the FPCs (T3–T5), *b* = 0.060, *p* = 0.033. The level of the risk subscale decreased faster from T3 to T5 for patients with cluster B diagnosis (*b* = −0.170, *p* < 0.001) compared to patients without this diagnosis (*b* = −0.111 *p* < 0.001; Figure 6). This hypothesis was tested by comparing a model with varying slopes for the two groups to a model with the same slopes. The results showed that the slopes were significantly different, Δ$\chi_{\left(1\right)}^{2}$ = 10.311, *p* < 0.001. It should be noted that we did not test differences in changes of protective awareness subscale over time between these two groups of patients, as this model did not fit the data well in this study and results should not be interpreted. Differences in risk and protective factors between cluster B and non-cluster B PDs patients considering each time point are displayed in Table 2.

Lastly, it was also tested whether SUD may influence trajectories of the clinical scale as well as the risk and protective subscales in a group of cluster B PDs patients. The results revealed no significant differences in these trajectories between cluster B PDs patients with SUD and those without.

## Discussion

The long-term changes of dynamic risk and protective factors have been rarely studied in forensic psychiatric patients. In addition, to our knowledge, no prior studies have examined whether these trajectories differ between patients depending on their psychiatric diagnosis, namely SUD, psychotic disorders, and cluster B PDs. Therefore, the main goal of this study was to investigate the changes in dynamic risk and protective factors over time utilizing LGCA in all male forensic psychiatric patients who were unconditionally released between 2004 and 2014 from one of the 12 Dutch FPCs. The period of investigation covered the entire stay in the FPC; from the moment of juridical observation until the moment of unconditional release. First, we tested the unconditional model for the clinical scale, as well as the risk, and protective subscales. Then, the conditional models were analyzed with SUD, psychotic disorders, and cluster B PDs as predictors. Finally, we tested whether changes in the clinical scale, and protective and risk subscales are influenced by SUD in psychotic and cluster B PDs patients. Overall, the results indicate that the rate of change of dynamic risk and protective factors is not constant over time and that there are some important differences in the pathways of these factors between SUD and non-SUD patients, psychotic and non-psychotic patients, as well as cluster-B and non-cluster B PDs patients. However, SUD did not modify the changes in risk and protective factors in psychotic and cluster B PDs patients.

### Changes Over Time of the Clinical Risk and Protective Factors: General Findings

Concerning our unconditional models, the results showed that changes in the severity score of the clinical scale and risk subscale follow a very similar two-phase linear pattern. That is, the score on the clinical scale and risk subscale significantly decreased over time, with the rate of change being greater in the first phase of the stay in the FPCs than in the second phase. Similarly, there was also a larger improvement on the level of the protective skills subscale in the first phase, while there was almost no progress on this subscale in the second phase. Our findings are in line with previous findings showing that dynamic risk factors together with the lack of protective factors, continuously decrease over the course of treatment (13, 15, 57). However, in previous research, this rate of change was constant throughout the treatment, while in our study it deviated from a simple linear trajectory and consisted of two linear phases. In other words, the rate of change was greater from admission to the FPC up to the moment of the first unguided leave, that is, when patients were allowed to leave the institution for a short period without guidance, than from the moment when patients went on unguided leave and onwards. These differences between the present findings and those obtained in previous studies can be attributed to very limited empirical evidence on this matter as well as a much larger sample of forensic psychiatric patients in our study.

It could be that progress was greater in the first phase of the stay because the most change of dynamic risk and protective factors is expected to occur at the beginning of treatment (58, 59). In addition, the present study further revealed that the moment when leave modalities were granted to patients for the first time could be seen as a ‘turning point’ in the treatment of offenders. Leave modalities play an important role in the treatment of offenders. The typical progression is from escorted to unescorted leave and finally to the unconditional release. All proposals for leave must be approved by the Ministry of Justice. During these leave modalities, patients are tested if they are able to take responsibility and apply the skills learned during the treatment. Unguided leave can be granted to patients only when staff members conclude there is no risk for reoffending and no immediate danger of a patient's escape (18). In line with this, our study showed that at this particular point, patients were indeed characterized by low scores on the clinical scale and the risk subscale, and high scores on the protective skills subscale. It may be that from the moment when the unguided leave was granted, patients did not need to change much during the rest of the treatment because the largest progress has already been made in the first phase of their stay.

### Patients With SUD vs. Patients Without SUD

Furthermore, we investigated if there are differences in trajectories of the clinical scale, and the risk and protective skills subscales between SUD and non-SUD patients, psychotic and non-psychotic patients and cluster B and non-cluster B PDs patients.

With regard to SUD, contrary to our expectations, the results showed no significant differences in the changes in the clinical scale and the risk subscale over time between patients with and without SUD. Even more unexpectedly, we found that SUD patients improved faster on the protective skills subscale than non-SUD patients, but only in the first phase of treatment, namely from the time of unguided leave to unconditional release. These findings may be attributable to contextual factors and the impact of forensic psychiatric treatment. Since our sample included forensic psychiatric patients staying in highly-secured FPCs, the potential for alcohol or illicit drugs may be significantly reduced than in the outside world, as these substances are not readily available for consumption in these institutions (i.e., there is strong control for their presence), thus forcing many patients to abstain (60). Hence it is plausible that the influence of SUD might be diminished. In addition, patients with SUD receive addiction treatment which usually starts with psychoeducation about substance use and increasing their intrinsic motivation. Furthermore, cognitive behavioral techniques are used to teach them prevention skills, such as helping thoughts to cope with urges and potentially risky situations (61, 62). The intervention has been proved to be efficient in resisting drug use (63), reducing maladaptive thinking (64), and decreasing self-reported substance use (65). This could be another explanation for non-significant differences regarding changes in the clinical scale and the risk subscale between SUD and non-SUD patients in this study. Moreover, the finding that SUD patients improved faster on protective skills than non-SUD patients in the first phase of their stay may be explained by the impact of the treatment. As mentioned, during the rehabilitation treatment, offenders with SUD learn different coping strategies in group settings, which may directly enhance their coping skills and perhaps indirectly their social skills. Therefore, this could be a reason for their faster improvement in protective skills overall compared to non-SUD patients. However, these differences were not significant in the second phase of the treatment, which could be attributed to the fact that treatment is more intensive in the first phase of their stay (58).

### Patients With Psychotic Disorders vs. Patients Without Psychotic Disorders

The findings showed that patients with psychotic disorders decreased faster on the clinical scale and risk subscale, and increased more strongly on the protective skills subscale than patients without these disorders, but only in the first phase of their stay in the FPCs. In the second phase of their stay, however, there were no significant differences in the change of these factors between the two groups of patients. Hence, our expectations that psychotic patients (compared to non-psychotic patients) would show less decrease in risk factors and less increase in protective factors over time are not supported. It is important to note, however, that a *post-hoc* ANOVA analysis showed that at the moment of juridical assessment (T1) and after the first 12 months of the stay in the FPCs (T2), psychotic patients scored significantly higher on the clinical scale, and the risk subscale, and significantly lower on the protective skills subscale than non-psychotic patients. This is consistent with evidence that individuals with a psychotic diagnosis are at higher risk for violence and criminal behavior than those without this diagnosis (17, 22, 31). That said, significant differences in trajectories of the clinical scale, and the risk and protective subscales in the first phase of the stay may be attributed to the differences in the initial levels of the risk and protective factors between psychotic and non-psychotic patients. This means that non-psychotic patients changed less on these factors because at the beginning of the treatment they displayed fewer risks and more protection against reoffending and hence did not need to change as much as psychotic patients. Alternatively, it might be that psychotic patients progressed more in the first phase of the treatment as a result of the received antipsychotic medications. The antipsychotic drugs produce structural changes in the brain, regulating its action (30). They can therefore also cause changes in risk and protective factors in psychotic patients.

However, during the second phase of their stay, no significant differences were detected in the risk and protective skills subscales between patients with and without psychotic disorders, except at the moment of conditional leave (T4). At this particular time point, psychotic patients scored significantly higher on the clinical scale. It may signify that there were some deviations from treatment progress in this group of patients at T4. Nevertheless, the present study shows that psychotic patients overall have benefited from forensic treatment, especially at the beginning of their stay in the institution. Therefore, the notion that patients with psychotic disorders are less responsive to treatment and more difficult to work with (33, 34) cannot be entirely supported by the findings of the present study. The somewhat contrasting finding could be explained by the fact that much has been done in recent years to improve treatment in forensic hospitals. For example, during their stay in the FPCs, patients are offered a wide range of treatment options, such as cognitive behavioral therapy, schema focus therapy, psychomotor therapy, music therapy, psychopharmaceutical therapy, and a combination of therapies (16). Hence, it may be that some of these options did indeed work well even for ‘difficult’ patients such as those with psychotic disorders.

Similarly, there were no significant differences in the trajectories of the clinical scale and the risk and protective subscales between psychotic patients with and without SUD comorbidity. As mentioned earlier, this might be due to the impact of the treatment and the fact that illicit drugs and alcohol are difficult (legally) accessible in high secure FPCs (60, 64). Therefore, it could be assumed that the use of these substances is reduced, which can subsequently benefit treatment progress.

### Patients With Cluster B PDs vs. Patients Without Cluster B PDs

Furthermore, we also found that cluster B PDs significantly predicted trajectories of the risk subscale, but only in the second phase of the patients' stay in the FPCs. That is, cluster B PDs patients decreased significantly faster from the moment of the unguided leave until unconditional release than non-cluster PDs B patients. This is not in line with our expectations that cluster B PDs patients would show less progress during treatment than non-cluster PDs B patients. In contrast, the results showed that cluster B PDs patients benefited from the treatment as well, especially from the moment of unguided leave onwards. In addition, the *post-hoc* ANOVA analysis further revealed that patients with cluster B PDs scored significantly higher on risk factors from T1 to T4, which corresponds with empirical evidence that cluster B PDs patients are overall at higher risk for criminal behavior [e.g., (43, 44)]. However, these differences were not significant anymore at the end of their stay in the FPC (T5), meaning that cluster B PDs patients completed treatment equally well as non-cluster B PDs patients. One possible reason for non-cluster B PDs patients showing less improvement in the second phase of the stay could be due to their overall lower risk for reoffending at the beginning of this phase, which also implies less necessity for change from that particular moment until the end of the treatment. Another reason could be that cluster B PDs patients showed greater improvement during that second phase because they might need more time to adjust to and comply with treatment's requirements once being admitted to the forensic hospital. In support of this, patients with cluster B are indeed deemed to be less likely to conform to social norms and rules, which in turn can lead to violations of terms and agreements as well as treatment non-adherence (23, 38). Alternatively, it could be that cluster B PDs patients displayed fake behavior and exaggerated their mental fitness to reduce mandatory treatment and obtain privileges, such as unguided leave (66). Faster improvement during the second phase of their stay could possibly result from increased motivation to deceive once they succeed in their intentions to obtain certain benefits, such as the first unguided leave. Previous research also showed that antisocial patients deploy under-reporting of symptoms and post-conviction social desirability to make a favorable impression on judicial decision makers while denying real problems, such as substance use and impulsivity (67).

In addition, we did not find significant differences between cluster B and non-cluster B PDs patients in the trajectories of the clinical scale, and the protective skills subscale. It could be speculated that differences were only found for the risk factors as they are more pronounced in patients with cluster B PDs (17, 23, 42–44) than the lack of protective factors (17, 68).

Finally, as in a group of psychotic patients, SUD did not influence the changes in the risk and protective factors in a group of cluster B PDs patients as well. Again, this finding could be explained by the effects of treatment for substance use and forced abstinence of patients during their stay in the FPCs (60, 64).

### Limitations, Suggestions for Future Research, and Clinical Implications

There may be some possible limitations in this study that could be addressed in future research. The first limitation concerns the use of the DSM-IV to diagnose and classify mental disorders instead of employing the latest edition of this manual, namely the DSM-5. However, at the time when this study was carried out, the DSM-5 had not yet been published. One of the key changes from DSM-IV to DSM-5 is the removal of the multiaxial system of diagnosis. Instead, the DSM-5 combines axes I to III into a single axis that depicts mental and other medical diagnoses. Nonetheless, it is likely that this does not affect the generalizability of our findings. The study was further limited by the fact that we did not control for the presence of other comorbid conditions than SUD in psychotic and cluster B PDs patients when examining group-specific trajectories of risk and protective factors, which may also affect the results (69). For example, in this sample 5.7 % (*n* = 41) patients had both psychotic disorder and cluster B PDs. Another limitation is that we did not have enough time points to identify a non-linear latent growth curve model for the protective awareness subscale, and thus to interpret it. Although two linear slopes were sufficient to capture the non-linear change of most dynamic risk and protective factors at scale and subscale levels, our findings indicate that when it comes to the protective awareness subscale, a piecewise model with at least three linear slopes might be necessary. However, this more complex three-slope model could not be tested as we only had five time points and this analysis requires at least seven time points for identification (56). Moreover, in all tested models, the slope variance was significant, indicating significant individual differences in the growth rates of the clinical scale and the risk and protective subscales. The same holds true even when we added predictors to the unconditional models. Future research should attempt to find which factors may explain these individual differences in the growth trajectories of dynamic risk and protective factors. Future studies may wish to consider examining these pathways between recidivists and non-recidivist as this could deepen our understanding of whether the rate of change may contribute to the relapse. Finally, our findings may not be generalizable to other international samples of high-risk forensic patients. Unlike in the Netherlands, in the United States, for example, most offenders suffering from PDs and/or SUD, are likely to end up in the prison system rather than in the forensic psychiatric institutions (18).

Despite these limitations, the findings from this study could be highly relevant to forensic mental health practitioners. Although at the end of the treatment the risk associated with reoffending was very low for all patients, our results showed that 118 (16.6 %) of them violently reoffended within 2 years after release. Of these, 48 (40.7%) were diagnosed with cluster B PDs of which 30 (25.4%) had comorbid SUD, and 27 (22.9%) were diagnosed with psychotic disorders of which 16 (13.6%) had comorbid SUD. This signifies that there is a need to further improve the effectiveness of treatment in forensic correctional facilities. This can be achieved, e.g., by improving the treatment of cluster B PDs patients in the first phase of their stay in the FPCs, especially in terms of reducing risk factors. Similarly, for psychotic and all other patients, more attention should be paid to improving the second phase of their treatment, since fewer changes usually tend to occur in that phase. Last but not least, our findings showed that not all patients follow the same growth rate, meaning that there is a lot of variability between them. This signifies that individualized treatment might be even preferred for some patients. Therefore, forensic mental health professionals may need to adapt the treatment for these patients in agreement with their learning style, motivation, abilities, and strengths (1, 5).

To sum up, the present study provides an insight into the change of the risk and protective factors over time in a highly representative sample of Dutch forensic psychiatric patients. Overall, findings suggest that all changes in dynamic risk and protective factors could be depicted by two phases of patients' stay in the FPCs. In addition, the moment of unguided leave could be considered as the ‘turning point’ in the treatment of offenders. Specifically, most changes in dynamic risk and protective factors occurred at the beginning of the treatment namely from the moment of juridical assessment up to the moment of the unguided leave. We also looked at group-specific long-term changes in these factors, and found that SUD patients and psychotic patients changed the most in the first phase of their stay, while cluster B PDs patients changed the most in the second phase. These findings may help improve offender treatment and crime prevention strategies. More effective treatment may lead to lower recidivism rates, better reintegration of offenders into society, and a safer environment for patients and others. However, the present study is not without limitations and our findings should only be considered preliminary. Future research is therefore necessary to replicate the findings of this study and to further investigate the effectiveness of treatment at different stages of the patient's stay in FPCs.

## Data Availability Statement

The raw data supporting the conclusions of this article will be made available by the authors, without undue reservation.

## Ethics Statement

The studies involving human participants were reviewed and approved by The Scientific Research Committee of the FPC Kijvelanden, the Dutch Ministry of Security and Justice, the directors of the FPCs involved in this study and the Ethical Review Board of Tilburg University, the Netherlands. In exceptional cases, research with patient file data is possible without permission (Article 7:458 paragraph 3 of the Dutch Civil Code [in Dutch: BW]).

## Author Contributions

MJ analyzed the data and wrote the first draft of the manuscript. GB, EM, EC, and SB critically revised the manuscript for important intellectual content. All authors contributed to and have approved the final manuscript.

## Conflict of Interest

The authors declare that the research was conducted in the absence of any commercial or financial relationships that could be construed as a potential conflict of interest.

## Publisher's Note

All claims expressed in this article are solely those of the authors and do not necessarily represent those of their affiliated organizations, or those of the publisher, the editors and the reviewers. Any product that may be evaluated in this article, or claim that may be made by its manufacturer, is not guaranteed or endorsed by the publisher.

## Footnotes

^1^From now on, we use the abbreviation FPC(s) to denote all these highly secured forensic psychiatric institutions.
