# Supplementary material for: The Long-Term Changes in Dynamic Risk and Protective Factors Over Time in a Nationwide Sample of Dutch Forensic Psychiatric Patients
Source: Front Psychiatry. 2021 Sep 16;12:737846. doi: 10.3389/fpsyt.2021.737846 (PMC8481688; doi:10.3389/fpsyt.2021.737846)
Supplement: Supplementary file 1 [file Table_1.DOCX]

Supplementary Material

# Supplementary Tables

Table S1

Description of Clinical Risk and Protective Factors

| Risk factors |  |
| --- | --- |
| K01: Psychotic symptoms | The extent to which the patient showed active psychotic symptoms such as delusions (e.g., hearing voices). Particular attention should be given to symptoms or delusions containing aspects of violence, paranoid content and/or with aspects of executing control over fear. |
| K02: Addiction | Includes alcohol, soft drugs (e.g., cannabis), hard drugs (e.g., heroin, cocaine, speed, LSD), and other addictions such as gambling, sex, internet, or medication. Considering the first three categories, assessment focus is on the frequency of the infringing consumption. If the controlled use of substances is part of the treatment, then it is not seen as infringing consumption. |
| K03: Impulsivity | The extent to which the patient behaved in an unpredictable and/or thoughtless way at the expense of himself or others (“first act, then think”). It can be expressed as irascibility (to have a short fuse), incontrollable immediate need-satisfaction desires (impulse-buys), or a chaotic manner of living. |
| K04: Antisocial behavior | The extent to which the patient pursued his own desires and needs without either taking others’ feelings and needs or the circumstances into account and the extent to which this resulted in transgressive behavior towards others. |
| K05: Hostility | The extent to which the patients attributed hostility to others, along with systems and authorities. Hostility can be manifested in the form of violent perceptions, passive aggression, cynicism, and annoyance, and sometimes in the form of severe verbal and physical aggression. |
| K06: Violation of terms and agreements | The extent to which the patient adhered to his terms and agreements of his treatment. This includes the severity as well as the frequency of potential breaches of treatment agreements, the institution’s rules, or furlough conditions. |
| K07: Influence by risky network members | The extent to which the patient allows himself to be influenced by others in a negative way, including fellow patients, family, friends etc. Risky networks can influence the patient to engage in risky situations or risky behavior. |
| Protective factors |  |
| K08: Problem insight | The extent to which the patient was aware of his individual risk factors and signals of risky behaviors in situations that can lead to his relapse, and the extent to which the patient behaves based on his awareness and knowledge. |
| K09: Social skills | The extent to which the patient was able to maintain social contact with his life- and work- environment in a satisfactory manner (e.g., communication skills, the proper use of manners, assertive behavior during contact with others, appropriate personal space during conversations, etc.). |
| K10: Self-reliance | The extent to which the patient was able to complete essential daily tasks independently, such as personal hygiene, dealing with money, patterns of diet, sleep patterns, self-presentation, care for his surroundings, and the ability to call for medical help. |
| K11: Treatment compliance | The extent to which the patient was involved in his treatment progression. It includes patient’s cooperation, participation during treatment components, the acceptance of rules, openness for other insights and coping strategies, and the intake of prescribed medication. |
| K12: Crime responsibility | The extent to which the patient accepts and takes responsibility for the offences he has committed. Some reasons for the denial of responsibility are, for instance, drug- or alcohol abuse, the fact that the offence may be committed by a group of people etc. |
| K13: Coping skills | The extent to which the patient showed the correct skills to successfully resolve a confrontation with the occurrences that require adaptations (e.g., talking calmly with someone, distancing oneself from the situation, asking for help etc.). |
| K14: Labor skills | The extent to which the patient was able to properly perform work or labor activities, such as work inside the clinic, a paid job, or a voluntary position with fixed tasks and hours. |

*Note*. Reprinted from “Longitudinal network structure and changes of clinical risk and protective factors in a nationwide sample of forensic psychiatric patients” by Bogaerts, S., Spreen, M., Masthoff, E., & Jankovic, M. (2020), International Journal of Offender Therapy and Comparative Criminology, 64(15).

Table S2

Results From the Factor Analysis of the Risk Subscale

|  | Component |
| --- | --- |
|  | Risk subscale |
| Violation of terms | .811 |
| Hostility | .786 |
| Impulsivity | .743 |
| Antisocial behavior | .723 |
| Psychotic symptoms | .633 |
| Influence by risky network members | .434 |
| Addiction | .104 |

Note. N = 722. The extraction method was principal component analysis with a varimax (Varimax with Kaiser Normalization) rotation.

Table S3

Results From the Factor Analysis of the Protective Subscale

|  | Component | |
| --- | --- | --- |
|  | Protective skills | Protective awareness |
| Social skills | .761 |  |
| Labor skills | .710 |  |
| Self-reliance | .695 |  |
| Coping skills | .694 |  |
| Responsibility for the offense |  | .826 |
| Problem insight |  | .773 |
| Treatment compliance |  | .629 |

Note. N = 722. The extraction method was principal component analysis with a varimax (Varimax with Kaiser Normalization) rotation.

Table S4

Skewness and Kurtosis for all the Variables

| Variable | Skewness | Kurtosis | *SE* |
| --- | --- | --- | --- |
| Clinical risk scale |  |  |  |
| T1 | 0.30 | -0.03 | 0.02 |
| T2 | 0.48 | -0.21 | 0.03 |
| T3 | 0.53 | -0.13 | 0.02 |
| T4 | 0.69 | 0.49 | 0.03 |
| T5 | 1.23 | 1.43 | 0.02 |
| Risk subscale |  |  |  |
| T1 | 0.47 | -0.28 | 0.03 |
| T2 | 0.62 | -0.21 | 0.03 |
| T3 | 0.78 | 0.11 | 0.02 |
| T4 | 0.89 | 0.49 | 0.03 |
| T5 | 1.57 | 2.36 | 0.02 |
| Protective awareness subscale |  |  |  |
| T1 | 0.64 | 0.16 | 0.03 |
| T2 | 0.10 | -0.86 | 0.04 |
| T3 | -0.50 | 0.53 | 0.03 |
| T4 | -0.45 | -0.54 | 0.04 |
| T5 | -1.16 | 0.91 | 0.03 |
| Protective skills subscale |  |  |  |
| T1 | -0.35 | 0.25 | 0.03 |
| T2 | -0.75 | 0.50 | 0.03 |
| T3 | 0.38 | 0.04 | 0.02 |
| T4 | -0.96 | 1.39 | 0.03 |
| T5 | -0.91 | 0.81 | 0.03 |

*Note*. *SE*=Standard error.
